# Learning analytics for smart campus: Data on academic performances of engineering undergraduates in Nigerian private university

**DOI:** 10.1016/j.dib.2017.12.059

**Published:** 2018-01-03

**Authors:** Segun I. Popoola, Aderemi A. Atayero, Joke A. Badejo, Temitope M. John, Jonathan A. Odukoya, David O. Omole

**Affiliations:** a Department of Electrical and Information Engineering, Covenant University, Ota, Nigeria; b Department of Psychology, Covenant University, Ota, Nigeria; c Department of Civil Engineering, Covenant University, Ota, Nigeria

**Keywords:** Smart campus, Learning analytics, Sustainable education, Nigerian university, Education data mining, Engineering

## Abstract

Empirical measurement, monitoring, analysis, and reporting of learning outcomes in higher institutions of developing countries may lead to sustainable education in the region. In this data article, data about the academic performances of undergraduates that studied engineering programs at Covenant University, Nigeria are presented and analyzed. A total population sample of 1841 undergraduates that studied Chemical Engineering (CHE), Civil Engineering (CVE), Computer Engineering (CEN), Electrical and Electronics Engineering (EEE), Information and Communication Engineering (ICE), Mechanical Engineering (MEE), and Petroleum Engineering (PET) within the year range of 2002–2014 are randomly selected. For the five-year study period of engineering program, Grade Point Average (GPA) and its cumulative value of each of the sample were obtained from the Department of Student Records and Academic Affairs. In order to encourage evidence-based research in learning analytics, detailed datasets are made publicly available in a Microsoft Excel spreadsheet file attached to this article. Descriptive statistics and frequency distributions of the academic performance data are presented in tables and graphs for easy data interpretations. In addition, one-way Analysis of Variance (ANOVA) and multiple comparison post-hoc tests are performed to determine whether the variations in the academic performances are significant across the seven engineering programs. The data provided in this article will assist the global educational research community and regional policy makers to understand and optimize the learning environment towards the realization of smart campuses and sustainable education.

**Specifications Table**

**Table:** 

Subject area	*Engineering Education*
More specific subject area	*Learning Analytics*
Type of data	*Tables, graphs, figures, and spreadsheet file*
How data was acquired	*For the five-year study period of engineering program, Grade Point Average (GPA) and its cumulative value of each of the sample were obtained from the Department of Student Records and Academic Affairs.*
Data format	*Raw, analyzed*
Experimental factors	*Undergraduates with incomplete academic records were excluded*
Experimental features	*Descriptive statistics, frequency distributions, one-way ANOVA and multiple comparison post-hoc tests were performed to determine whether the variations in the academic performances are significant across the seven engineering programs.*
Data source location	*The population sample and the academic performance data provided in this article were obtained at Covenant University, Canaanland, Ota, Nigeria (Latitude 6.6718*^*o*^*N, Longitude 3.1581*^*o*^*E)*
Data accessibility	*In order to encourage evidence-based research in learning analytics, detailed datasets are made publicly available in a Microsoft Excel spreadsheet file attached to this article.*

**Value of the data**

• Comprehensive academic performance datasets provided in this article will promote evidence-based research in the emerging field of learning analytics in developing countries [1], [2], [3], [4].
• Easy access to this data will assist the global educational research community and regional policy makers to understand and optimize the learning environment towards the realization of smart campuses and sustainable education [5], [6], [7], [8], [9], [10].
• With the growing adoption of machine learning and artificial intelligence techniques in different fields, empirical data provided in this article will help in the development of predictive models for learning outcomes in engineering undergraduates [11], [12], [13], [14], [15], [16], [17], [18].
• Descriptive statistics, frequency distributions, one-way ANOVA and multiple comparison post-hoc tests that are presented in tables, plots, and graphs will make data interpretation much easier for useful insights and logical conclusions.
• Detailed datasets that are made publicly available in a Microsoft Excel spreadsheet file attached to this article will encourage further explorative studies in this field of research.

## 1 Data

The emerging field of learning analytics may be exploited to improve learning outcomes of engineering undergraduates in higher institutions of developing countries towards attaining sustainable education in the region [19], [20], [21]. Useful information about the academic performances of undergraduates that studied engineering programs at Covenant University, Nigeria are presented and analyzed in this data article. Covenant University is located in Ota, Ogun State in Nigeria *(Latitude 6.6718*^*o*^ *N, Longitude 3.1581*^*o*^ *E)*. It is a private Christian university affiliated with Living Faith Church Worldwide and a member of the Association of Commonwealth Universities (ACU), Association of African Universities (AAU), and National Universities Commission (NUC).

A total population sample of 1841 undergraduates that studied Chemical Engineering (CHE), Civil Engineering (CVE), Computer Engineering (CEN), Electrical and Electronics Engineering (EEE), Information and Communication Engineering (ICE), Mechanical Engineering (MEE), and Petroleum Engineering (PET) within the year range of 2002–2014 are randomly selected. The earliest year of entry and the latest year of graduation are 2002 and 2014 respectively. Having excluded undergraduates with incomplete academic records, 198, 152, 374, 407, 349, 166, 195 undergraduates were pooled from CHE, CVE, CEN, EEE, ICE, MEE, and PET respectively. The descriptive statistics of the academic performances of undergraduates in each of the seven engineering programs at Covenant University are presented in Table 1, Table 2, Table 3, Table 4, Table 5, Table 6, Table 7.

**Table 1:** Descriptive statistics of academic performances of undergraduates in CHE.

	*First Year GPA*	*Second Year GPA*	*Third Year GPA*	*Fourth Year GPA*	*Fifth Year GPA*	*Cumulative GPA*
Mean	4.02	3.49	3.52	3.77	3.79	3.70
Median	4.11	3.53	3.55	3.88	3.90	3.78
Mode	4.15	2.74	3.13	4.06	4.43	3.73
Standard Deviation	0.57	0.69	0.77	0.79	0.67	0.61
Variance	0.32	0.48	0.59	0.63	0.45	0.37
Kurtosis	4.07	2.69	2.40	2.70	3.45	2.39
Skewness	−0.97	−0.34	−0.33	−0.64	−0.85	−0.36
Range	2.82	3.24	3.47	3.42	3.41	2.70
Minimum	2.09	1.54	1.47	1.55	1.59	2.16
Maximum	4.91	4.78	4.94	4.97	5.00	4.86
Total Samples	198	198	198	198	198	198

**Table 2:** Descriptive statistics of academic performances of undergraduates in CVE.

	*First Year GPA*	*Second Year GPA*	*Third Year GPA*	*Fourth Year GPA*	*Fifth Year GPA*	*Cumulative GPA*
Mean	3.67	3.13	3.33	3.78	3.91	3.54
Median	3.70	3.09	3.38	3.92	4.01	3.60
Mode	4.02	3.14	2.76	4.17	4.89	3.76
Standard Deviation	0.60	0.69	0.85	0.74	0.71	0.65
Variance	0.36	0.47	0.72	0.54	0.50	0.42
Kurtosis	3.48	2.55	2.28	2.24	2.60	2.27
Skewness	−0.47	0.25	−0.15	−0.42	−0.57	−0.06
Range	3.36	3.22	3.94	3.03	3.15	2.96
Minimum	1.60	1.70	0.99	1.94	1.83	1.97
Maximum	4.96	4.92	4.93	4.97	4.98	4.93
Total Samples	152	152	152	152	152	152

**Table 3:** Descriptive statistics of academic performances of undergraduates in CEN.

	*First Year GPA*	*Second Year GPA*	*Third Year GPA*	*Fourth Year GPA*	*Fifth Year GPA*	*Cumulative GPA*
Mean	3.61	3.23	3.38	3.64	3.62	3.50
Median	3.71	3.22	3.51	3.72	3.68	3.56
Mode	4.00	3.20	4.47	4.07	4.25	3.21
Standard Deviation	0.71	0.76	0.90	0.77	0.72	0.69
Variance	0.50	0.58	0.81	0.59	0.52	0.48
Kurtosis	2.58	2.50	2.36	3.33	2.73	2.44
Skewness	−0.43	0.03	−0.43	−0.61	−0.45	−0.24
Range	3.20	3.74	4.01	4.40	3.55	3.10
Minimum	1.73	1.19	0.97	0.60	1.39	1.80
Maximum	4.93	4.93	4.98	5.00	4.94	4.90
Total Samples	374	374	374	374	374	374

**Table 4:** Descriptive statistics of academic performances of undergraduates in EEE.

	*First Year GPA*	*Second Year GPA*	*Third Year GPA*	*Fourth Year GPA*	*Fifth Year GPA*	*Cumulative GPA*
Mean	4.03	3.49	3.60	3.54	3.58	3.66
Median	4.11	3.48	3.73	3.57	3.64	3.71
Mode	4.13	3.22	3.96	3.48	4.00	3.28
Standard Deviation	0.56	0.73	0.83	0.76	0.74	0.66
Variance	0.31	0.54	0.69	0.58	0.55	0.43
Kurtosis	3.07	2.50	2.56	2.59	2.49	2.43
Skewness	−0.61	−0.17	−0.55	−0.38	−0.32	−0.29
Range	3.23	3.56	3.95	3.69	3.58	3.05
Minimum	1.71	1.34	1.05	1.31	1.42	1.83
Maximum	4.94	4.90	5.00	5.00	5.00	4.88
Total Samples	407	407	407	407	407	407

**Table 5:** Descriptive statistics of academic performances of undergraduates in ICE.

	*First Year GPA*	*Second Year GPA*	*Third Year GPA*	*Fourth Year GPA*	*Fifth Year GPA*	*Cumulative GPA*
Mean	3.56	3.18	3.30	3.58	3.74	3.47
Median	3.55	3.18	3.36	3.62	3.82	3.51
Mode	3.49	3.06	3.02	3.52	4.00	3.51
Standard Deviation	0.69	0.76	0.88	0.73	0.71	0.68
Variance	0.48	0.57	0.77	0.54	0.50	0.46
Kurtosis	2.57	2.42	2.32	2.66	2.72	2.44
Skewness	−0.33	0.06	−0.24	−0.40	−0.48	−0.16
Range	3.32	3.49	3.89	3.49	3.23	3.09
Minimum	1.64	1.39	1.09	1.51	1.75	1.80
Maximum	4.96	4.88	4.98	5.00	4.98	4.89
Total Samples	349	349	349	349	349	349

**Table 6:** Descriptive statistics of academic performances of undergraduates in MEE.

	*First Year GPA*	*Second Year GPA*	*Third Year GPA*	*Fourth Year GPA*	*Fifth Year GPA*	*Cumulative GPA*
Mean	3.92	3.33	3.13	3.60	3.78	3.54
Median	4.00	3.32	3.04	3.73	3.96	3.57
Mode	4.00	3.69	3.13	4.55	4.30	3.95
Standard Deviation	0.60	0.72	0.87	0.76	0.73	0.66
Variance	0.36	0.52	0.76	0.58	0.54	0.43
Kurtosis	3.12	2.19	2.06	2.74	2.70	2.25
Skewness	−0.69	0.03	0.05	−0.57	−0.67	−0.14
Range	2.67	3.32	3.58	3.72	3.25	2.89
Minimum	2.20	1.55	1.40	1.25	1.73	1.99
Maximum	4.87	4.87	4.98	4.97	4.98	4.88
Total Samples	166	166	166	166	166	166

**Table 7:** Descriptive statistics of academic performances of undergraduates in PET.

	*First Year GPA*	*Second Year GPA*	*Third Year GPA*	*Fourth Year GPA*	*Fifth Year GPA*	*Cumulative GPA*
Mean	3.86	3.24	3.32	3.54	3.71	3.54
Median	3.91	3.18	3.33	3.54	3.75	3.56
Mode	3.78	2.48	3.74	3.61	3.20	3.83
Standard Deviation	0.62	0.71	0.73	0.69	0.65	0.59
Variance	0.38	0.50	0.54	0.48	0.42	0.35
Kurtosis	3.83	2.54	2.46	2.67	2.39	2.43
Skewness	−0.88	−0.04	−0.15	−0.03	−0.18	−0.01
Range	3.29	3.74	3.64	3.55	2.83	2.73
Minimum	1.64	1.22	1.18	1.45	2.13	2.07
Maximum	4.93	4.96	4.82	5.00	4.95	4.80
Total Samples	195	195	195	195	195	195

**Fig. 1:**
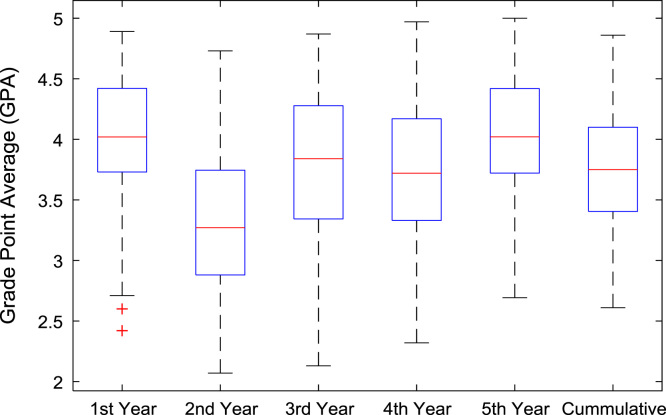
Boxplot of GPA data of undergraduates in the seven engineering programs (2002–2014).

**Fig. 2:**
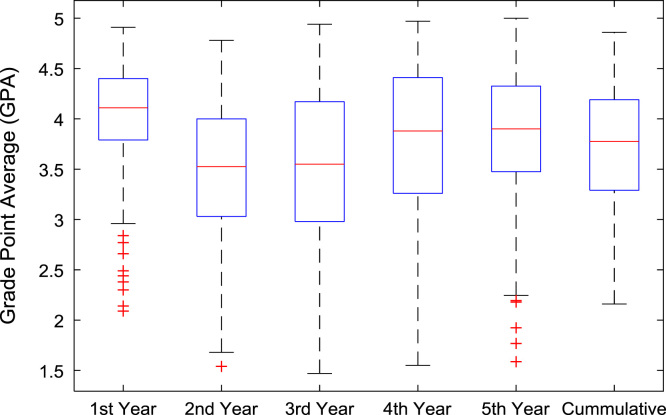
Boxplot of GPA data of undergraduates in CHE (2002–2014).

**Fig. 3:**
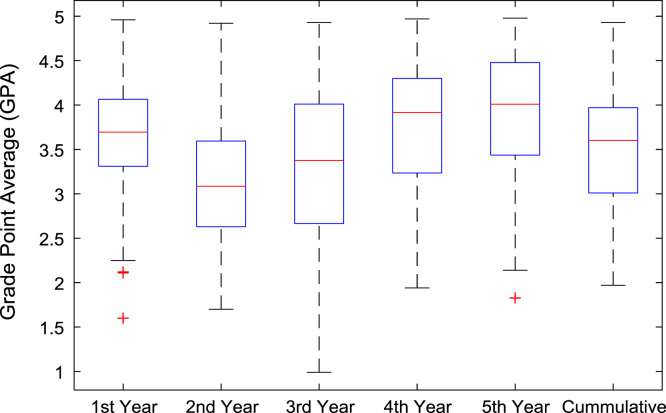
Boxplot of GPA data of undergraduates in CVE (2002–2014).

**Fig. 4:**
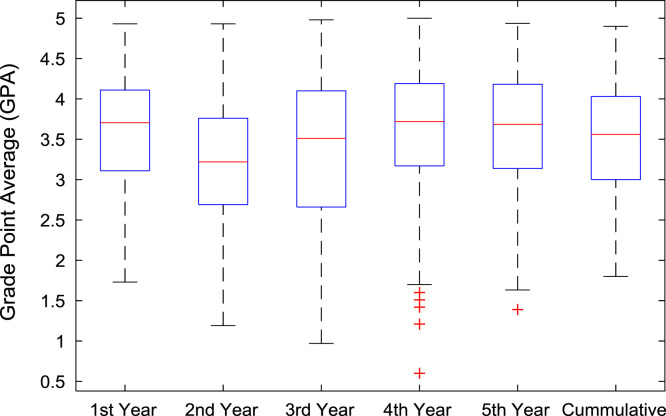
Boxplot of GPA data of undergraduates in CEN (2002–2014).

**Fig. 5:**
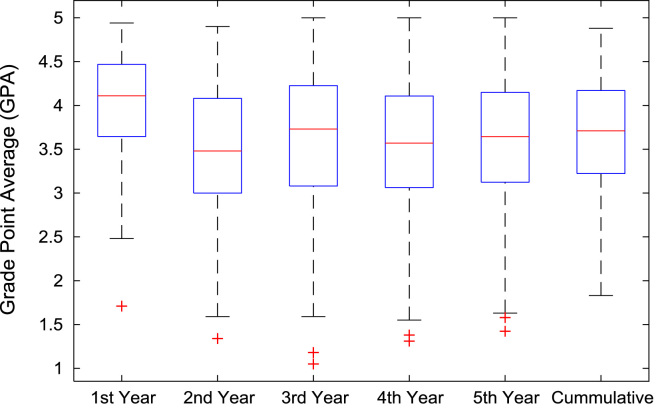
Boxplot of GPA data of undergraduates in EEE (2002–2014).

**Fig. 6:**
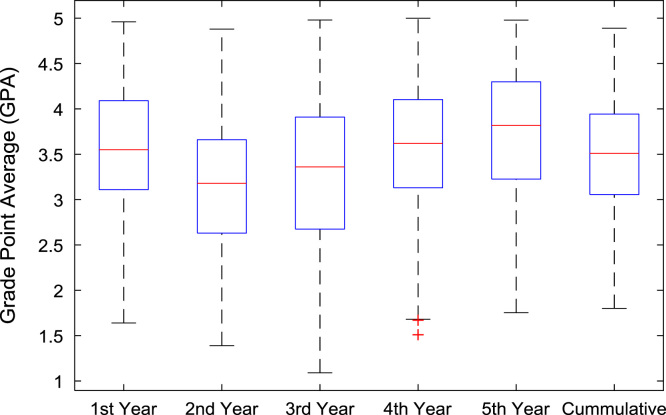
Boxplot of GPA data of undergraduates in ICE (2002–2014).

**Fig. 7:**
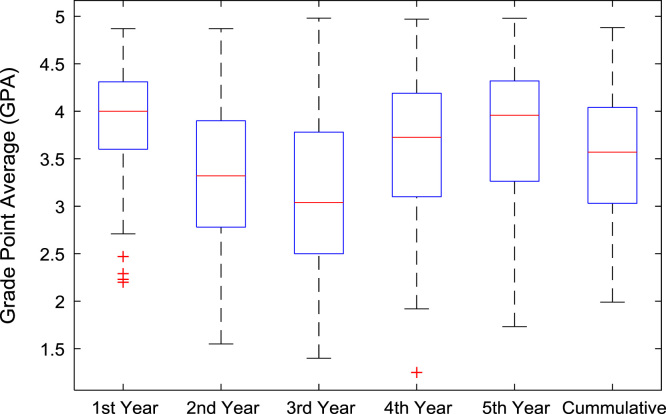
Boxplot of GPA data of undergraduates in MEE (2002–2014).

**Fig. 8:**
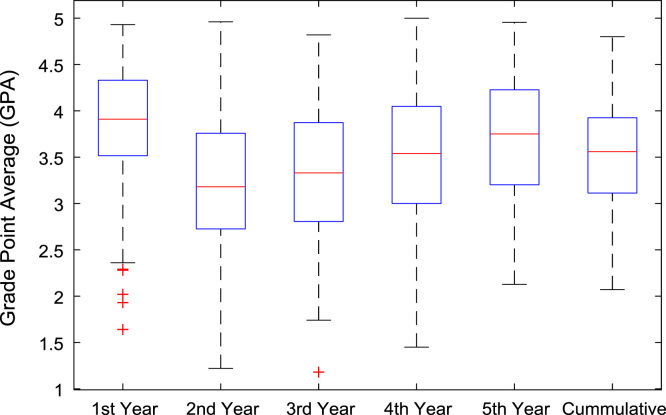
Boxplot of GPA data of undergraduates in PET (2002–2014).

**Fig. 9:**
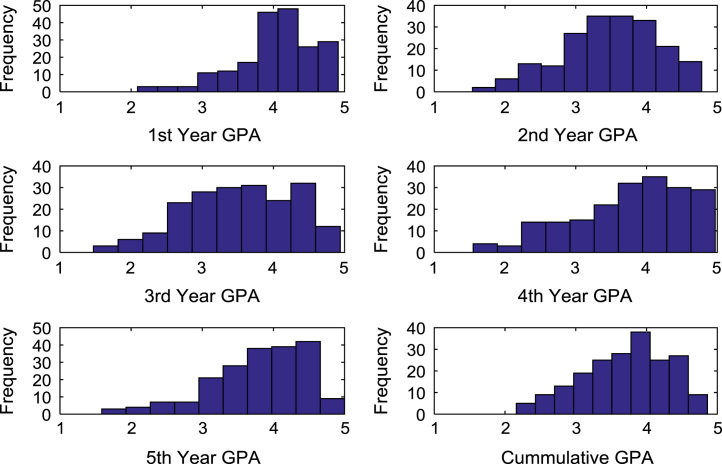
Histogram distributions of GPA data of undergraduates in CHE.

**Fig. 10:**
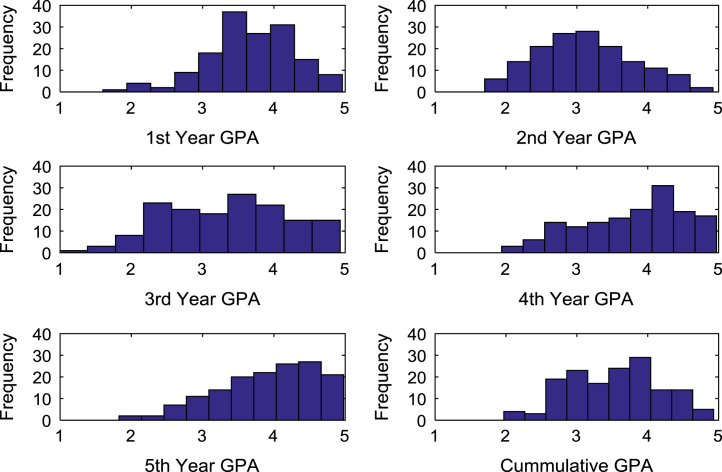
Histogram distributions of GPA data of undergraduates in CVE.

**Fig. 11:**
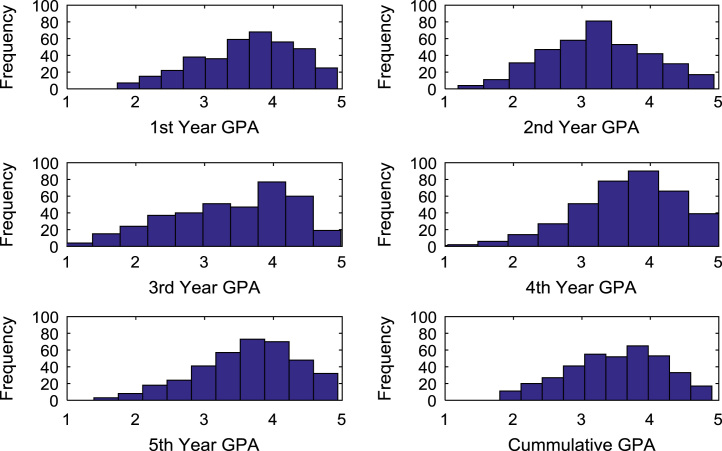
Histogram distributions of GPA data of undergraduates in CEN.

**Fig. 12:**
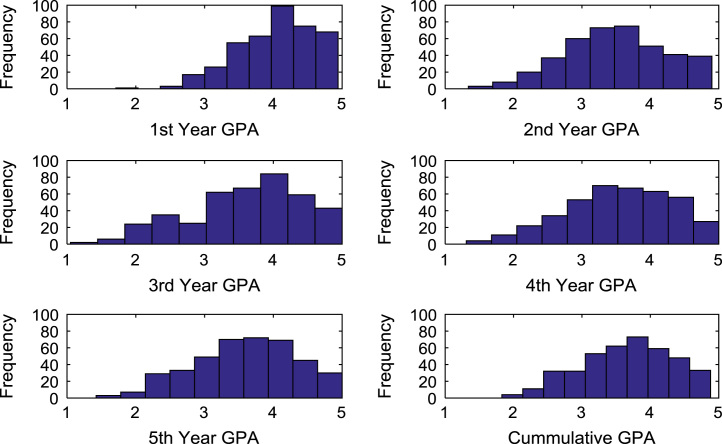
Histogram distributions of GPA data of undergraduates in EEE.

**Fig. 13:**
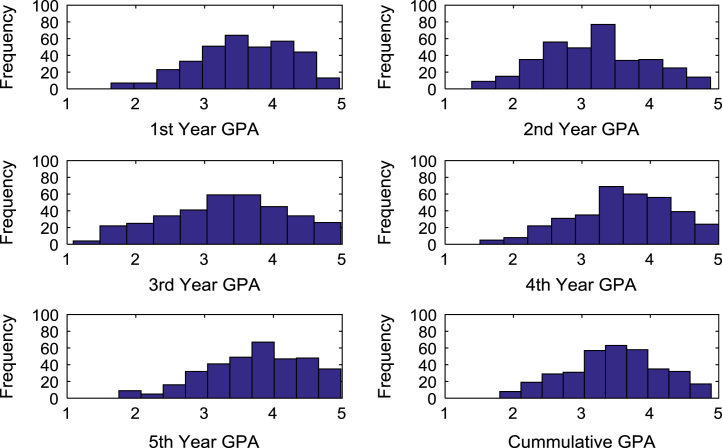
Histogram distributions of GPA data of undergraduates in ICE.

**Fig. 14:**
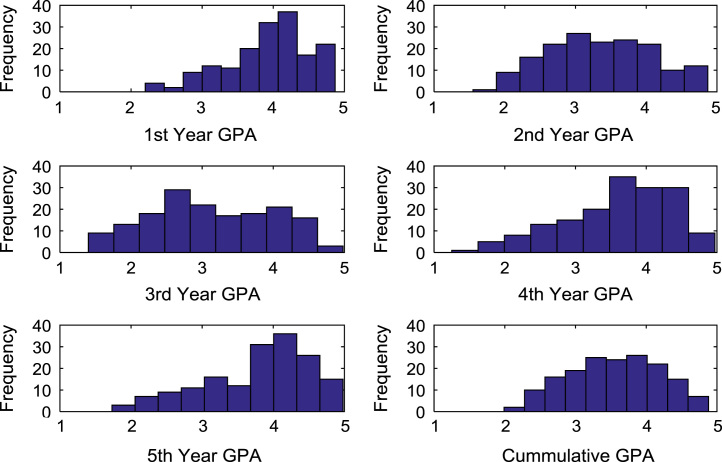
Histogram distributions of GPA data of undergraduates in MEE.

**Fig. 15:**
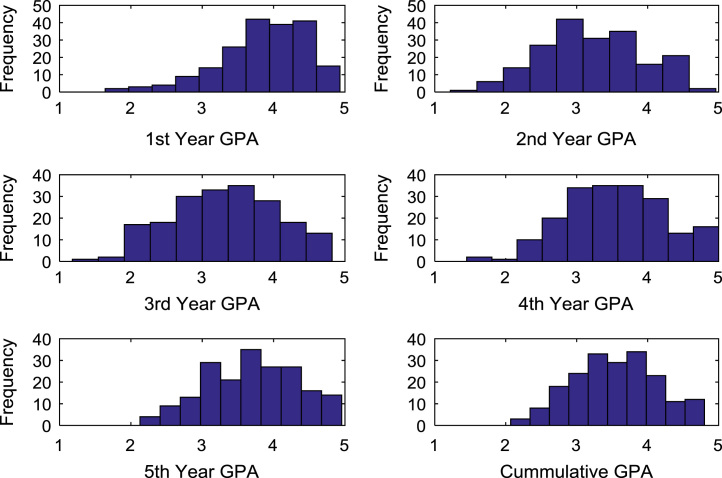
Histogram distributions of GPA data of undergraduates in PET.

**Fig. 16:**
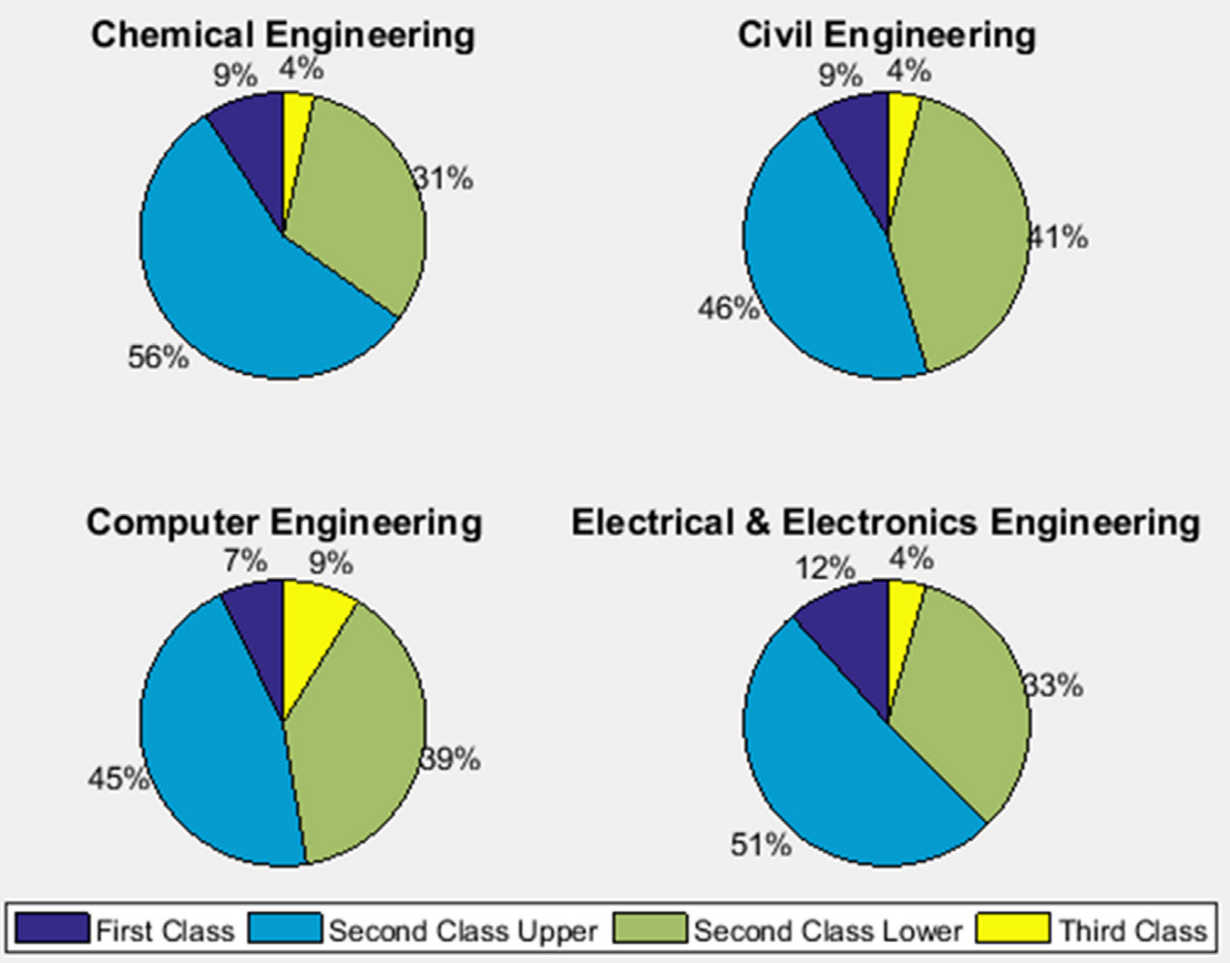
Proportions of class of degree in CHE, CVE, CEN, and EEE.

**Fig. 17:**
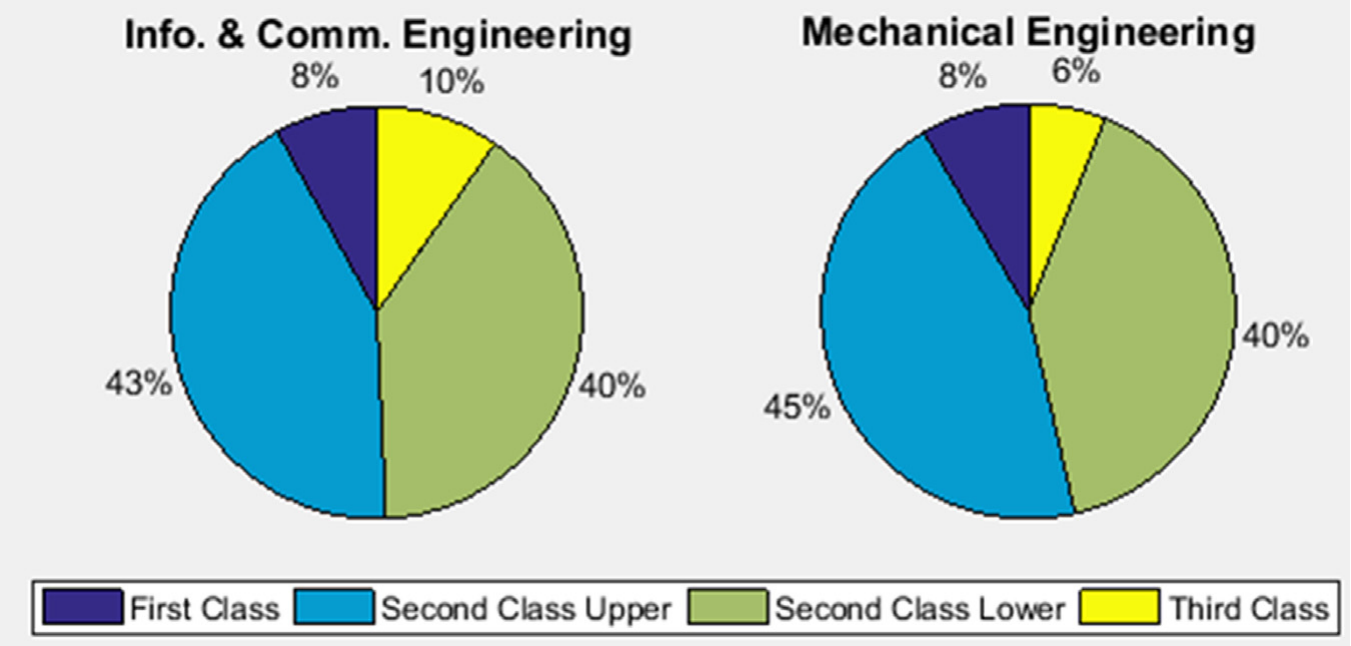
Proportions of class of degree in ICE and MEE.

**Fig. 18:**
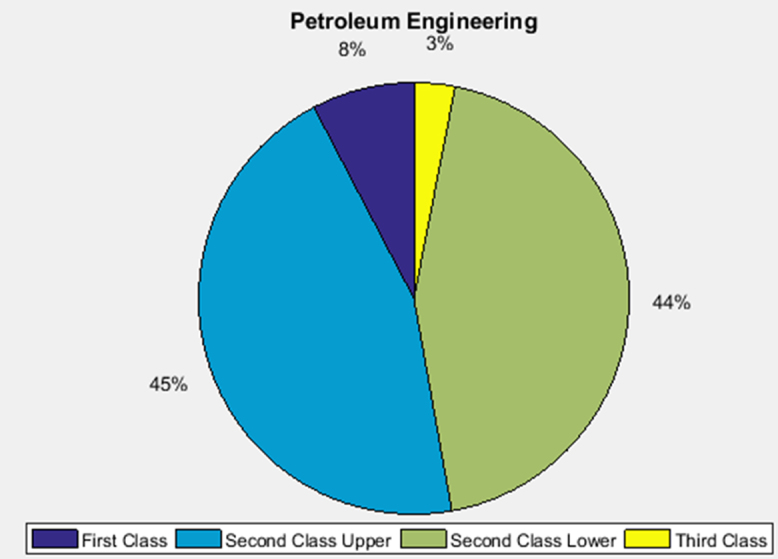
Proportions of class of degree in PET.

The academic performances of engineering undergraduates vary as the students proceed from one level to another yearly. Fig. 1 shows the variations in the GPA data of all the engineering undergraduates under investigation. Fig. 2, Fig. 3, Fig. 4, Fig. 5, Fig. 6, Fig. 7, Fig. 8 illustrate the differences and trends in the GPA data of undergraduates in CHE, CVE, CEN, EEE, ICE, MEE, and PET respectively. The frequency distributions of the GPA data of undergraduates in CHE, CVE, CEN, EEE, ICE, MEE, and PET are shown in Fig. 9, Fig. 10, Fig. 11, Fig. 12, Fig. 13, Fig. 14, Fig. 15 respectively. Fig. 16, Fig. 17, Fig. 18 depict the proportions of engineering students that graduated with First Class, Second Class Upper, Second Class Lower, and Third Class in CHE, CVE, CEN, and EEE; ICE and MEE; and PET respectively.

## 2 Experimental design, materials and methods

**Fig. 19:**
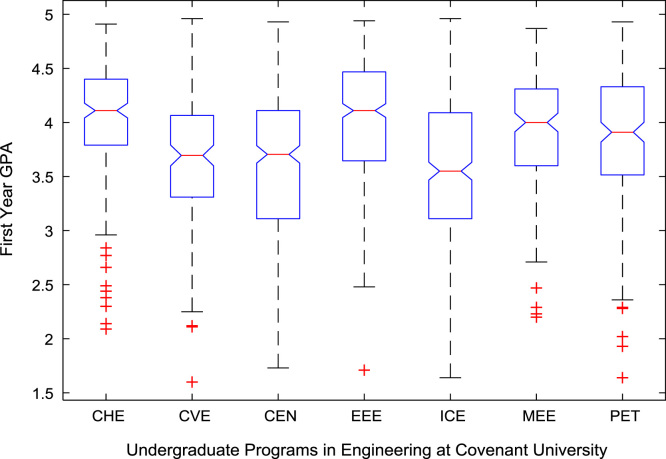
First year GPA data of all engineering programs.

**Fig. 20:**
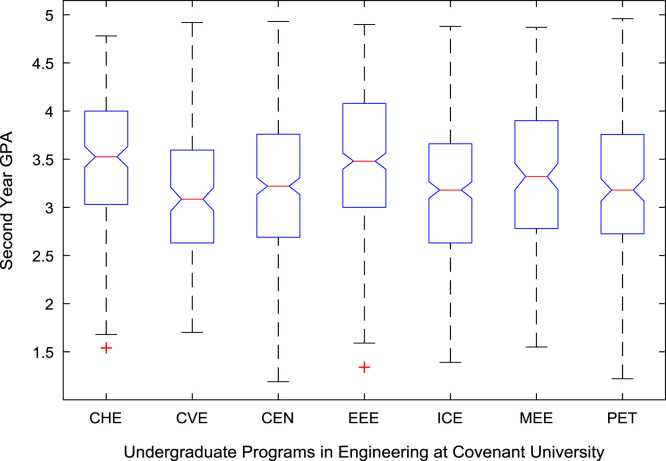
Second year GPA data of engineering programs at Covenant university.

**Fig. 21:**
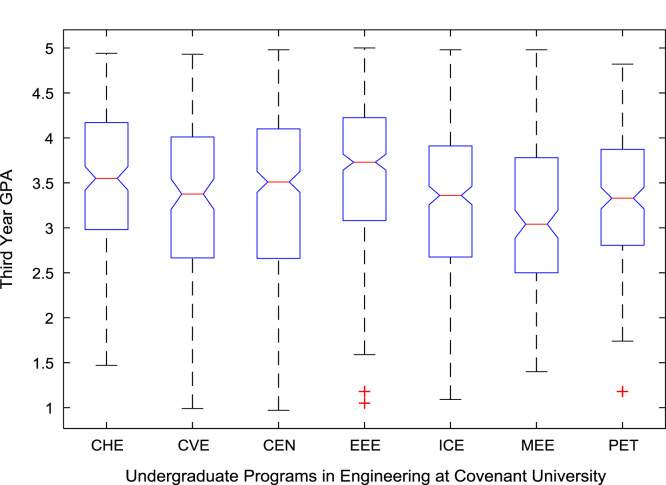
Third year GPA data of engineering programs at Covenant university.

**Fig. 22:**
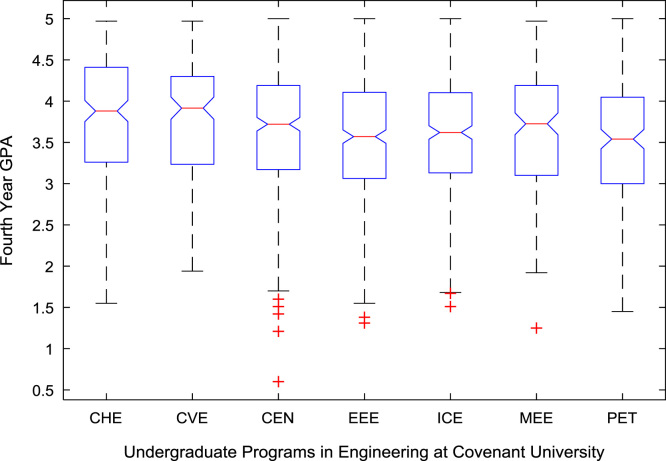
Fourth year GPA data of engineering programs at Covenant university.

**Fig. 23:**
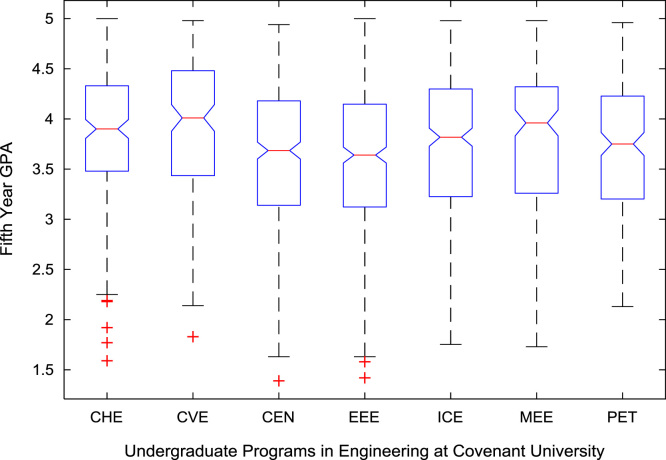
Fifth year GPA data of engineering programs at Covenant university.

**Fig. 24:**
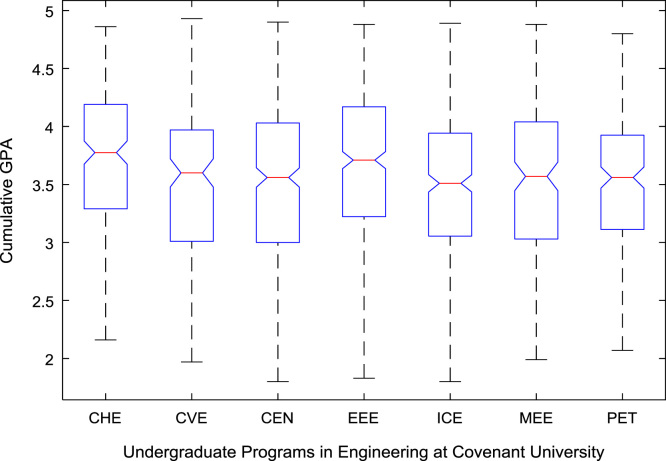
Cumulative GPA data of engineering programs at Covenant university.

**Fig. 25:**
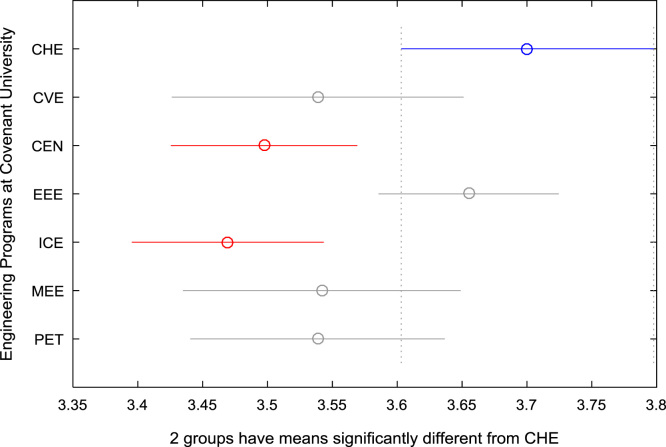
Multiple comparison test on cumulative GPA for CHE.

**Fig. 26:**
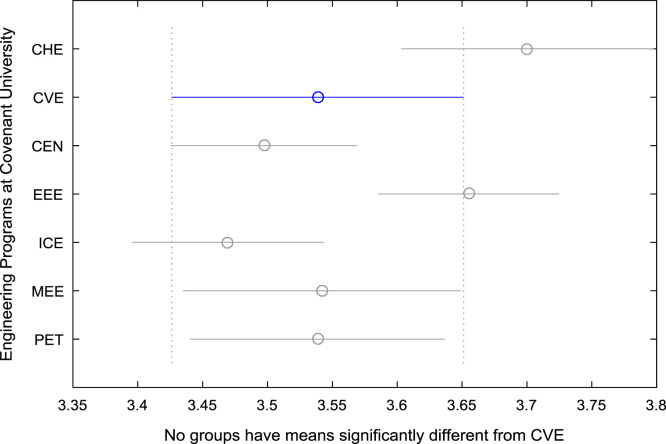
Multiple comparison test on cumulative GPA for CVE.

**Fig. 27:**
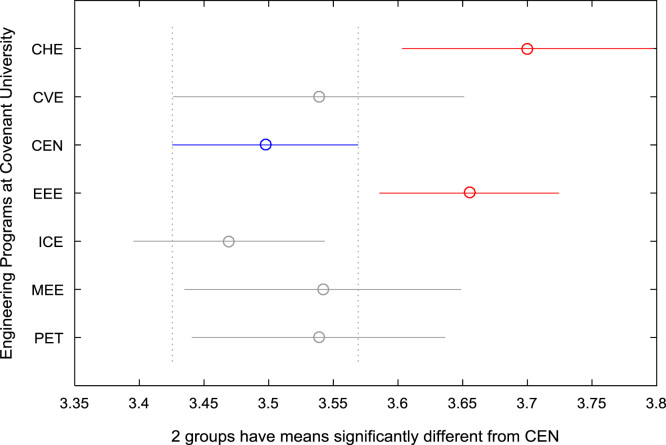
Multiple comparison test on cumulative GPA for CEN.

**Fig. 28:**
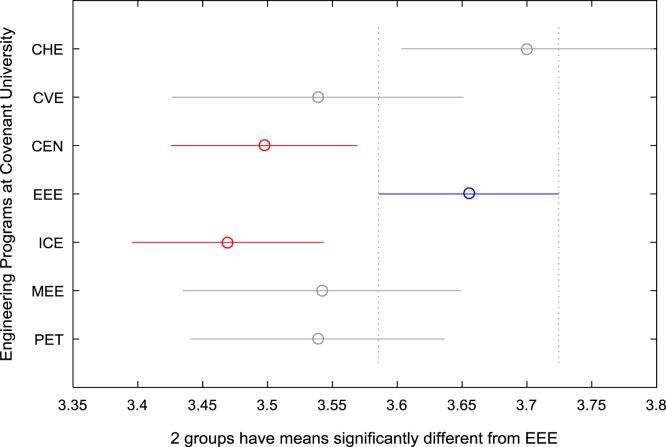
Multiple comparison test on cumulative GPA for EEE.

**Fig. 29:**
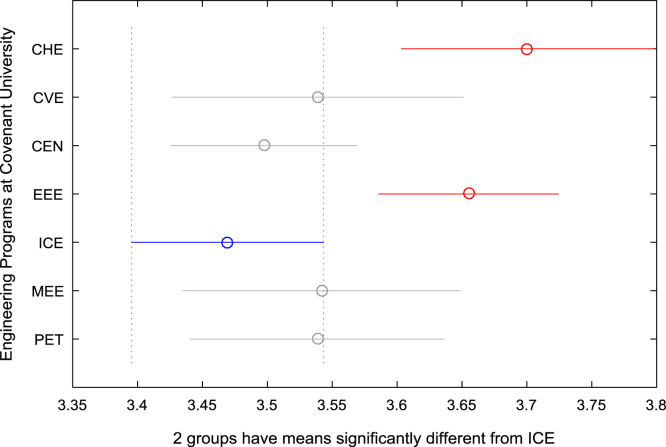
Multiple comparison test on cumulative GPA for ICE.

**Fig. 30:**
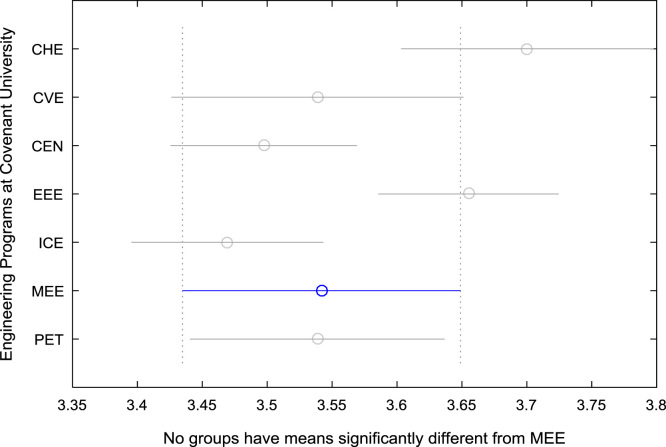
Multiple comparison test on cumulative GPA for MEE.

**Fig. 31:**
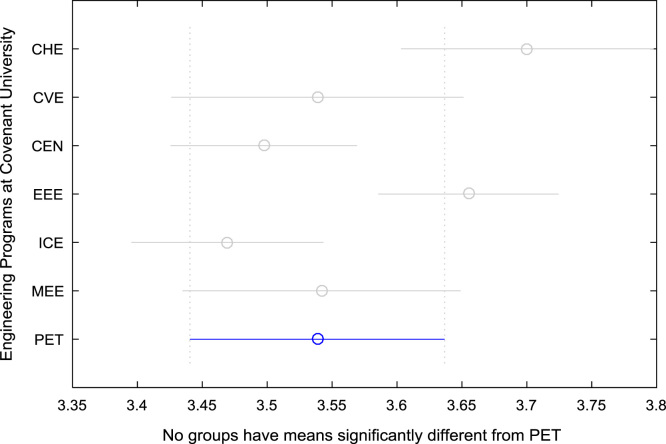
Multiple comparison test on cumulative GPA for PET.

For the five-year study period of engineering program, Grade Point Average (GPA) and its cumulative value of each of the sample were obtained from the Department of Student Records and Academic Affairs. In order to encourage evidence-based research in learning analytics, detailed datasets are made publicly available in a Microsoft Excel spreadsheet file attached to this article. Descriptive statistics and frequency distributions of the academic performance data are presented in tables and graphs for easy data interpretations. In addition, one-way Analysis of Variance (ANOVA) and multiple comparison post-hoc tests are performed to determine whether the variations in the academic performances are significant across the seven engineering programs. Data showing whether there are significant differences in the GPA data of the engineering undergraduates throughout their five-year study period are presented in Table 8, Table 9, Table 10, Table 11, Table 12, Table 13. The boxplots of the GPA distribution by program are shown in Fig. 19, Fig. 20, Fig. 21, Fig. 22, Fig. 23, Fig. 24. The results of the post-hoc test conducted to understand the extent of significant variations in cumulative GPA across engineering Programs at Covenant University are presented in Table 14. Multiple comparison plots of Cumulative GPA data in Fig. 25, Fig. 26, Fig. 27, Fig. 28, Fig. 29, Fig. 30, Fig. 31 reveal groups (i.e. other engineering programs at Covenant University) whose statistical means are significantly different.

**Table 8:** ANOVA test on first year GPA data of engineering programs at Covenant university.

Source of variation	Sum of squares	Degree of freedom	Mean squares	F Statistic	Prob>F
Columns	69.15	6	11.52	28.95	2.99×10^–33^
Error	730.21	1834	0.40		
Total	799.36	1840

**Table 9:** ANOVA test on second year GPA data of engineering programs at Covenant university.

Source of variation	Sum of squares	Degree of freedom	Mean squares	F statistic	Prob>F
Columns	34.02	6	5.67	10.58	1.43×10^–11^
Error	983.13	1834	0.54		
Total	1017.15	1840

**Table 10:** ANOVA test on third year GPA data of engineering programs at Covenant university.

Source of variation	Sum of squares	Degree of freedom	Mean squares	F statistic	Prob>F
Columns	36.48	6	6.08	8.55	3.47×10^-9^
Error	1304.02	1834	0.71		
Total	1340.51	1840

**Table 11:** ANOVA test on fourth year GPA data of engineering programs at Covenant university.

Source of variation	Sum of squares	Degree of freedom	Mean squares	F statistic	Prob>F
Columns	12.99	6	2.16	3.83	8.53×10^-4^
Error	1037.83	1834	0.57		
Total	1050.82	1840

**Table 12:** ANOVA test on fifth year GPA data of engineering programs at Covenant university.

Source of variation	Sum of squares	Degree of freedom	Mean squares	F statistic	Prob>F
Columns	17.80	6	2.97	5.87	4.44 × 10^-6^
Error	926.63	1834	0.51		
Total	944.43	1840

**Table 13:** ANOVA test on cumulative GPA data of engineering programs at Covenant university.

Source of variation	Sum of squares	Degree of freedom	Mean squares	F statistic	Prob>F
Columns	12.13	6	2.02	4.70	9.39×10^-5^
Error	789.25	1834	0.43		
Total	801.38	1840

**Table 14:** Post-hoc test on cumulative GPA for engineering programs at Covenant university.

Groups compared		Lower limits for 95% confidence intervals	Mean difference	Upper limits for 95% confidence intervals	*p*-value
CHE	CVE	−0.0469	0.1617	0.3703	0.2507
CHE	CEN	0.0331	0.2031	0.3731	0.0078
CHE	EEE	−0.1222	0.0453	0.2129	0.9853
CHE	ICE	0.0590	0.2310	0.4031	0.0015
CHE	MEE	−0.0450	0.1585	0.3621	0.2455
CHE	PET	−0.0333	0.1618	0.3570	0.1798
CVE	CEN	−0.1447	0.0414	0.2274	0.9948
CVE	EEE	−0.3002	−0.1164	0.0675	0.5029
CVE	ICE	−0.1186	0.0693	0.2573	0.9321
CVE	MEE	−0.2203	−0.0032	0.2139	1.0000
CVE	PET	−0.2091	0.0001	0.2094	1.0000
CEN	EEE	−0.2963	−0.1577	−0.0192	0.0139
CEN	ICE	−0.1160	0.0280	0.1719	0.9976
CEN	MEE	−0.2249	−0.0445	0.1358	0.9909
CEN	PET	−0.2121	−0.0412	0.1296	0.9919
EEE	ICE	0.0446	0.1857	0.3268	0.0020
EEE	MEE	−0.0649	0.1132	0.2913	0.4979
EEE	PET	−0.0520	0.1165	0.2849	0.3898
ICE	MEE	−0.2549	−0.0725	0.1099	0.9047
ICE	PET	−0.2421	−0.0692	0.1037	0.9020
MEE	PET	−0.2009	0.0033	0.2076	1.0000

## References

[1] Protonotarios V., Stoitsis G., Kastrantas K., Sanchez-Alonso S. (2013). Using multilingual analytics to explore the usage of a learning portal in developing countries. J. Asynchronous Learn. Netw..

[2] R. Ferguson, A. Cooper, H. Drachsler, G. Kismihók, A. Boyer, K. Tammets, et al., Learning analytics: European perspectives, in: ACM International Conference Proceeding Series, pp. 69–72, 2015.

[3] C. Gavan, Developing a framework for the effective use of learning analytics: a UK perspective, in Student Engagement and Participation: Concepts, Methodologies, Tools, and Applications. vol. 1, ed. pp. 369–398, 2017.

[4] J. Halliday, M. Anderson, Developing a framework for the visualisation of learning analytics in UK higher education, in: Decision Management: Concepts, Methodologies, Tools, and Applications. vol. 1–4, ed. pp. 249–250, 2017.

[5] Johnston T. (1992). Population, education and sustainable development. Afr. Dev. Rev..

[6] Nicolas A., Radja K., Schembri P. (2009). Which education for a sustainable development in developing countries? An approach by competencies. Mondes En. Dev..

[7] A. Roy, P. Kihoza, J. Suhonen, M. Vesisenaho, Promoting education for sustainable development by using ICT enhanced problem based learning in a developing country, in: Proceedings of the 4th International Conference on Technology for Education, IEEE, T4E 2012, pp. 98–104, 2012.

[8] S. Munoz-Hemandez, Looking for sustainable software for education in developing countries, in: Proceedings of the IEEE Global Engineering Education Conference, EDUCON, pp. 1108–1111, 2014.

[9] W.A. Segura, Education and sustainable development. The challenge for developing countries to change paradigms, in: Proceedings of the 10th International Multi-Conference on Society, Cybernetics and Informatics, IMSCI, pp. 194–199, 2016.

[10] Nguyen T.P. (2017). Education for sustainable development in Vietnam: exploring the geography teachers' perspectives. Int. Res. Geogr. Environ. Educ..

[11] D. Gibson, J. Clarke-Midura, Some psychometric and design implications of game-based learning analytics, in: Proceedings of the IADIS International Conference on Cognition and Exploratory Learning in Digital Age, CELDA, pp. 201–208, 2013.

[12] D. Gasevic, A. Wolff, C. Rose, Z. Zdrahal, G. Siemens, Learning analytics and machine learning, in: Proceedings of the ACM International Conference Series, pp. 287–288, 2014.

[13] D. Gibson, S.D. Freitas, Exploratory learning analytics methods from three case studies, in: Proceedings of ASCILITE 2014 - Annual Conference of the Australian Society for Computers in Tertiary Education, pp. 383–388, 2014.

[14] J. Zheng, A. Dagnino, An initial study of predictive machine learning analytics on large volumes of historical data for power system applications, in: Proceedings of the International Conference on Big Data, IEEE Big Data, pp. 952–959, 2014.

[15] N. Brouwer, B. Bredeweg, S. Latour, A. Berg, G. van der Huizen, Learning analytics pilot with coach2 - Searching for effective mirroring, in: Lecture Notes in Computer Science (including subseries Lecture Notes in Artificial Intelligence and Lecture Notes in Bioinformatics) vol. 9891 LNCS, ed. pp. 363–369, 2016.

[16] Petkovic D. (2016). Using learning analytics to assess Capstone project teams. Computer.

[17] J. Gardner, C. Brooks, Statistical approaches to the model comparison task in learning analytics, in: Proceedings CEUR Workshop, 2017.

[18] M.J. Junokas, G. Kohlburn, S. Kumar, B. Lane, W.T. Fu, R. Lindgren, Using one-shot machine learning to implement real-time multimodal learning analytics, in: Proceedings CEUR Workshop, pp. 89–93, 2017.

[19] Ayo C.K., Odukoya J.A., Azeta A. (2014). A review of open & distance education and human development in Nigeria. Int. J. Emerg. Technol. Learn..

[20] Odukoya J.A., Adekeye O., Igbinoba A.O., Afolabi A. (2017). Item analysis of university-wide multiple choice objective examinations: the experience of a Nigerian private university. Qual. Quant..

[21] Odukoya J.A., Adekeye O., Okunlola O. (2017). Assessing the effectiveness of mobile learning devices in tertiary institutions: the experience of undergraduates in a Nigerian Private University. Int. J. Interact. Mob. Technol..

